# Reconstructing Protein Structures by Neural Network Pairwise Interaction Fields and Iterative Decoy Set Construction

**DOI:** 10.3390/biom4010160

**Published:** 2014-02-10

**Authors:** Claudio Mirabello, Alessandro Adelfio, Gianluca Pollastri

**Affiliations:** 1 School of Computer Science and Informatics, University College Dublin, Belfield, Dublin 4, Ireland; 2 Complex and Adaptive Systems Laboratory, University College Dublin, Belfield, Dublin 4, Ireland

**Keywords:** protein folding, protein structure prediction, artificial neural networks

## Abstract

Predicting the fold of a protein from its amino acid sequence is one of the grand problems in computational biology. While there has been progress towards a solution, especially when a protein can be modelled based on one or more known structures (templates), in the absence of templates, even the best predictions are generally much less reliable. In this paper, we present an approach for predicting the three-dimensional structure of a protein from the sequence alone, when templates of known structure are not available. This approach relies on a simple reconstruction procedure guided by a novel knowledge-based evaluation function implemented as a class of artificial neural networks that we have designed: Neural Network Pairwise Interaction Fields (NNPIF). This evaluation function takes into account the contextual information for each residue and is trained to identify native-like conformations from non-native-like ones by using large sets of decoys as a training set. The training set is generated and then iteratively expanded during successive folding simulations. As NNPIF are fast at evaluating conformations, thousands of models can be processed in a short amount of time, and clustering techniques can be adopted for model selection. Although the results we present here are very preliminary, we consider them to be promising, with predictions being generated at state-of-the-art levels in some of the cases.

## 1. Introduction

Predicting the fold of a protein from its amino acid sequence is one of the grand open problems in computational biology [1], called protein structure prediction or protein folding prediction, with the former having a slightly broader connotation. In the scheme of protein structure prediction, two main groups of techniques have long being identified, whose applicability depends on the characteristics of the protein itself. If this belongs to a fold that has already been observed, it is often possible to use a *Homology Modelingor Template-Based*(TB) approach. If the right fold can be identified, by a variety of techniques ranging from simple sequence-sequence alignments to more complex approaches relying on structural information, knowledge- or physics-based potentials and, sometimes, elaborate statistical learning algorithms [2,3,4,5,6,7,8,9], it is generally possible to predict at least the core of a structure with good approximation by using one or more known structures belonging to the fold as “templates”. Some domains at the latest CASPcompetitions were predicted by TB techniques with very high accuracy [10,11,12] (up to 95% of the protein within one Ångstrom of the native).

There is, however, the case of proteins whose fold has not been observed thus far, also known as *Novel Folds*. In this case, predicting via TB is not an option, as there are no known templates. Moreover, even when a protein does in fact belong to a known fold, it is possible that current techniques may not be able to identify it. This is often the case when the relationship between the protein and the known instances of the correct fold is one of very remote homology or of analogy. In order to predict the structure of such proteins, models have to be built only starting from the amino acid chain, without any help from the templates. This is the case of *de novo*, *or ab initio* (AI) modeling. When modelling is attempted by AI techniques, the results are generally much poorer than in the TB case [12,13], as we are not fully capable of mimicking nature in a reliable manner. Normally, methods attempting to predict proteins *ab initio* try to fold a protein conformation under the guidance of an energy function, be this physics-based or knowledge-based (or, most often, a combination of the two). Both the manner of folding, the energy function and their interplay come in a dazzling variety of forms [14,15,16,17,18,19,20,21,22], although it is clear that full atomistic treatment of a protein comes at a tremendous cost and is feasible only in very limited circumstances on currently available hardware [23].

Although the fraction of proteins for which TB techniques are applicable has steadily grown for years, partly because of the growth of the Protein Data Bank [24], partly because of the impact of structural genomics initiatives [25], a sizeable fraction of known sequences still stubbornly belongs to the AI category, and improved AI techniques are going to be necessary for the foreseeable future.

Folding proteins by AI techniques remains both interesting and challenging. A comprehensive solution to the problem would give a better understanding of how proteins actually work and behave *in vivo* and may lead to the ability to design proteins *in silico*. The challenges of this approach, however, are highlighted by the fact that a solution has remained elusive in spite of decades of efforts.

The core of the problem is that replicating the way nature works is computationally costly, way beyond what we can conceivably afford, and as a consequence, the AI problem has to be solved by simplified models, which trade off accuracy for computational tractability.

In this paper, we present the prototype of a new approach to AI protein folding: a Protein Model Reconstructorthat uses a potential function based on a class of artificial neural networks we have designed [26]. This potential function, which measures the native-likeness of a protein conformation, is based on a very simple (hence, computationally affordable) geometrical model of a protein, but is also capable of evaluating the local context of each of the residues in the protein and, especially, is adaptive and requires minimal human intervention: rather than starting from a physical model and shedding complexity or designing a complex feature set to base the evaluation of a conformation upon, we simply train the model to identify native-like or partially native-like conformations from non-native-like ones by using a large set of decoys as a training set. We model this potential function by adapting a fast, easily scalable Protein Model Quality Assessment Program(MQAP) based on Neural Network-Pairwise Interaction Fields (NNPIF) [26]. This model inputs the conformation as a set of coordinates and amino acid identities and takes care of automatically extracting a feature set that guides its evaluation. We have implemented and describe in this work an original protocol in which training decoys are iteratively generated by the NNPIF themselves during successive folding simulations, in what is a mix of supervised and self-taught learning. NNPIF is capable of evaluating a protein model in a fraction of a second by only relying on its alpha carbon atoms (or *C_α_* trace) as the input, along with a small number of other simple features, and no information from structural templates is needed. As a result of this, thousands of models can be evaluated in a short amount of time, and clustering techniques can be adopted for model selection.

Although the results we present here are preliminary, we consider them to be promising, with predictions being generated at state-of-the-art levels in some of the cases.

## 2. Experimental Section

### 2.1. Overview

This ab *initio* predictor can be divided in two main blocks:

- A protein structure (*C_α_* trace) reconstructor;
- A neural network predicting the quality of a model, serving as an evaluation/potential function, that guides the reconstructor.

The model reconstructor is a component that manipulates a set of 3D coordinates representing a protein conformation, or model. While a complete protein structure model contains coordinates for every atom in the backbone and side-chains of the protein, this particular model reconstructor only uses a simplified representation of the protein, where each residue is represented by a single point, coinciding with its *C_α_* atom. This minimal set of coordinates is generally called the *C_α_* trace.

A model reconstruction is carried over a number of steps, where the *C_α_* trace is progressively shaped in a fold as close as possible to the perfect (native) one. In order to do so, the native-likeness of the *C_α_* trace has to be evaluated by an evaluation function, so that the reconstruction leads to building a good final model.

In this case, the evaluation function is a knowledge-based potential based on a parametric, adaptive algorithm, an artificial neural network (ANN) called the “Neural Network-Pairwise Interaction Field” (NNPIF) [26], which we have developed. An NNPIF automatically maps the interaction between each *C_α_* atom with its closest neighbour atoms into a feature vector. This feature vector is hidden, that is, it is learned with the aim of being most informative to predict the quality of the protein conformation. All hidden features are computed for all of the *C_α_* atoms and added up into a global feature vector for the conformation. This global feature vector is mapped via a second processing stage of the NNPIF into a global target property, which, in this case, is the quality (native-likeness) of the conformation.

The reconstructor implements the modelling process as a random search in which local perturbations to the *C_α_* trace are generated, and the resulting conformation is ranked by the evaluation function and kept or abandoned according to a probabilistic schedule.

The NNPIF evaluation function needs to be trained on examples (often called “decoys”) for which some meaningful measure of native-likeness is known. In fact, one such measure needs to be adopted: ideally, one that may be easily calculated for a conformation for which the native structure is known and that is, at the same time, meaningful, and that induces a tractable optimisation landscape. In this work, we use a measure of native-likeness that is purely geometrical, and gauge the overall distance between a conformation and its native state essentially by assessing the match between native contacts and contacts in the conformation, where a contact is simply a pair of *C_α_* atoms that are within a predefined threshold in the Euclidean space.

Even when a measure of native-likeness can be easily obtained, sampling the vast space of protein conformations (even when simplified as *C_α_* traces) is difficult, especially when one does not know beforehand where the evaluation function will guide the search. In other words, a decoy set may represent very well a part of the conformational space, and the evaluation function trained on this decoy set may, thus, evaluate conformations from that part of the space satisfactorily, but this is not much use if the search leads elsewhere.

In order to deal with this problem, instead of using a static training decoy set, new examples are generated and added to the training set as training proceeds. That is, if the evaluation function leads the search in a part of the conformational space, that part is automatically sampled, and the samples are added to the decoy set. More specifically, training is split in a number of different phases. The training set is initialised with native structures from the Protein Data Bank (PDB) [24]. Then, before the *n*-th training phase starts, more examples are generated by sampling the search pathways followed by the reconstructor using the NNPIF trained in phase *n* − 1 and added to the training set. The *n*-th training phase will then employ the new, expanded dataset. The same procedure is iterated until the desired training depth is reached. While this is essentially supervised learning, it borrows some ideas from self-teaching, e.g., [27], and active learning [28].

In order to gauge the accuracy of the NNPIF-based predictor, we perform tests using two different sets containing a number of target proteins (targets). As the predictor can produce a single model in a short amount of time, many different models are generated and grouped in clusters based on their mutual similarity, as the biggest clusters tend to contain models that are closer to the native one [29].

A small number of models is selected from the clusters and compared to the native model for the target. From the comparison, we obtain a score for the quality of the model called the Global Distance Test(GDT) score. The GDT score is described in [30] as:

(1) $$\text{GDT}(\text{predicted},\;\text{native})=\left({\text{GDT}}_{1}+{\text{GDT}}_{2}+{\text{GDT}}_{4}+{\text{GDT}}_{8}\right)/4$$

where *GDT_X_* is the percentage of atoms of the predicted model that are closer than *X* Å to their homologous in the native model when the two structures are superimposed.

### 2.2. NNPIF

The NNPIF considers the interactions between couples of amino acids (AA) in a model in order to evaluate its quality, or native-likeness. Only the *C_α_* trace of a model is taken into account. A version of this NNPIF has been used in the past as a model quality assessment system (MQAP) [26], which is a similar problem, in that a protein conformation has to be evaluated as a whole and mapped into a measure of its quality.

The NNPIF uses two stages, implemented as layered feed-forward neural networks (FFNN), to predict the native-likeness of a model based on its *C_α_* trace. In order to use an FFNN model, the input (in this case, the protein conformation) needs to be represented as a directed acyclic graph (DAG) [26,31,32,33]). While a conformation can be naturally viewed as an undirected graph in which atoms are nodes and interactions are edges, there are numerous ways to encode this representation in a DAG form [33,34,35,36].

**Figure 1:**
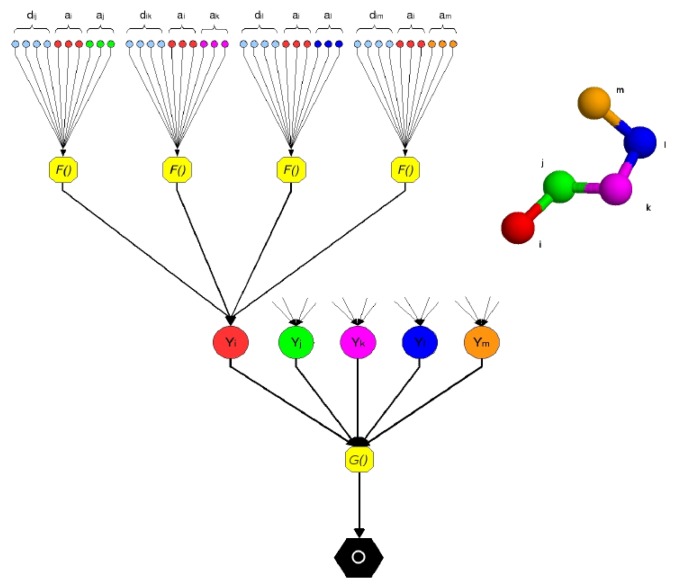
A pentapeptide structure is processed by the Neural Network Pairwise Interaction Fields (NNPIF). The structure is represented as a directed acyclic graph (DAG) by considering as leaf nodes the interactions between couples of atoms. Each interaction is modeled by the function, *F*(), via an NN, *N^F^*. The inputs encode characteristics of each of the two interacting atoms (*a_x_*, *a_y_*) in the pair and their interaction (*d_xy_*). The outputs are combined in hidden vectors *Y*. The contribution of each interaction to the quality of the structure is modeled by the function, *G*(), via an NN, *N^G^*, which produces the global output, *O*.

In this work, in order to represent a protein structure as a DAG, we consider the interactions between *C_α_* atoms as leaves of a tree where the root is the output of the predictor (Figure 1).

The interaction between two *C_α_* atoms (*i*, *j*) is described as follows:

(2) $$X_{ij}=F\left(a_{i},a_{j},d_{ij}\right)$$

where *a_i_* and *a_j_* are vectors describing the two interacting atoms in position *i* and *j* in the sequence and *d_ij_* describes the nature of the interaction between them. Notice how this representation is by no means limited to describing the atoms' identity and their distance. For instance, *a_i_* can be used to represent an AA's *C_α_* by the identity of the AA it is in alongside the identities of several AAs that neighbour it in the sequence or in a conformation. Evolutionary information extracted from similar sequences predicted the structural features, such as secondary structure, *etc*. Similarly, *d_ij_* is generally a vector containing an encoding of any pairwise property of atoms *i* and *j*, which is considered relevant for the prediction. This will naturally include the distance between the atoms, but may also describe the type of covalent bond between the atoms, if any, the possible presence of a hydrogen bond between the AA the atoms are part of, *etc*. Notice also how, given the flexibility of the input encoding (any unary or binary property of atoms can be represented), more mediated information could easily be added, for instance, correlated mutations, which have proven valuable for the prediction of protein residue contacts [37].

Function *F*() is implemented by an FFNN, *N^F^*, with a single hidden layer and a sigmoidal (hyperbolic tangent) output. This function is computed for each *C_α_* atom, *i*, considering only the 10 closest neighbours in the Euclidean space with the sequence separation distance >5. On average, a residue is within a 20 Å distance from 20 other residues, including the ones with sequence separation distance < 5 [38]. If we call the set of the 10 neighbours to the *i* − *th* atom Ci, we can compute the feature vector for *a_i_*:

(3) $$Y_{i}=K\sum_{j\in C_{i}}X_{ij}$$

Where *K* is a normalization constant. Notice that this feature vector is in fact the sum of the outputs of the hidden layers of 10 neural networks. That is, it has no explicit meaning, and no direct supervision is necessary for it; but, upon success in training, it will be a representation of the complex of all the interactions between an AA and its close neighbours, which is locally optimal for the prediction of the overall target property (the conformation's native-likeness).

The feature vectors for each of the *C_α_* atoms in the trace are computed and then combined (added component by component) to produce a global feature vector. This vector is input to the second layer of the NNPIF, which directly predicts the target property:

(4) $$O=G\left(\sum_{i=1}^{L}Y_{i}\right)$$

where *L* is the length of the protein. The function, *G*(), is implemented by a FFNN *N^G^* with a single hidden layer and a linear output.

The functions, *F* and *G*, are considered stationary (the same functions can be used, respectively, for each pair of atoms and for each conformation); so, the same *N^F^* is replicated for all of the interactions, and the same *N^G^* is used for all of the models.

Training of the overall NNPIF is performed by gradient descent, using the squared distance between the predicted and the target evaluation value. The gradient is computed in closed form, via a version of the back-propagation algorithm [39].

To speed up training, we do not compute the gradient of the error on all the training set, but estimate it on a small number of examples. This allows a speedier convergence, as many gradient descent steps are made for each evaluation of the training set instead of only one. Moreover, the stochastic element introduced by this practice (as the error landscape is estimated and not exact, it varies at every learning step) is helpful in avoiding local minima in the optimisation. In our experiments, the gradient is computed once for every two examples that are evaluated. Considering an average of 60 AAs per conformation, *N^F^*'*s* weights are updated once every 1,200 interactions that are evaluated, while *N^G^*'*s* weights are updated once every two contributions to its gradient that are calculated.

The NNPIF can evaluate a single model in a fraction of a second of CPU time. This means that thousands of models can be generated in a reasonable amount of time. Training, on the other end, can take weeks or months of computing time to complete, depending on the size of the training set used.

#### 2.2.1. Inputs, Output

The interactions between couples of neighbouring *C_α_* atoms are considered as the input.

For each atom, *i*, in the *C_α_* trace, an ordered list, *L_i_*, of other atoms in the *C_α_* trace is compiled. The order of a given atom, *j*, in the list *L_i_* is given by the Euclidean distance between *i* and *j*, *D*_ij_; the closest atom will be the first in the list, the farthest will be the last. All of the atoms that have a sequence separation distance with *i* <=5 are removed from the list. The first 10 atoms in the list are then selected as neighbours of *i*.

Considering atom *i*, for each of the neighbours, *j*, the inputs to the NN are:

- Identities, predicted secondary structure (predicted *ab initio* by Porter [40,41,42]) and the predicted solvent accessibility (predicted *ab initio* by PaleAle [41,42]) for the residues [*i* − 1, *i* + 1], [*j* − 1, *j* + 1] and the cosine of the corresponding angle between consecutive virtual bonds between *C_α_s*. Given the vector of coordinates *C*[*i*] for the residue i, we have (*C*[*i*] − *C*[*i* − *1*])*x*(*C*[*i* + 1] −*C*[*i*])(*C*[*i* + 2] − *C*[*i* + 1]);
- Euclidean distance between all pairs in [*i* − 1, *i* + 1], [*j* − 1, *j* + 1].

The output, *O*, for the second layer of the NNPIF is the predicted sum of recall and precision of the Residue Contact Map [43] for the input model. Precision and recall both range from 0 to 1, but the sum is rescaled so that the output ranges from −1 to 1, 1 corresponding to a perfect model (perfect superimposition with the native structure), −1 to a completely wrong one.

### 2.3. *C_α_* Trace Reconstructor

The *C_α_* trace reconstructor manipulates a model by changing the coordinates of a set of *C_α_* atoms at each step of the reconstruction procedure. This is done by extracting a fragment (*snippet*) of the model and replacing it with a different fragment. Snippets are extracted from high quality models from the Protein Data Bank by a process briefly sketched below.

First, the primary structure for the target is processed by our 1D protein feature predictors (Porter [40,41,42], PaleAle [41,42], BrownAle [7,44], Porter+ [7,45]). The predicted 1D features are then combined in a structural fingerprint identical to the one used in [46] and described in [7]. A series of profile-profile alignments then are run against a redundancy reduced PDB database. This way, for each target protein, we obtain a database of templates. Notice that this being an *ab initio* system, we do not perform such a search in order to find templates that bear a resemblance with the native conformation of our target. Rather, we just split the templates in structural fragments of 9 consecutive residues that will compose the snippet library.

Using the snippets insures that the resulting structures are locally accurate. If the secondary structure is correctly predicted, this can also help in finding a model with a correct super-secondary structure.

Given the *i* − *th* AA in the target protein, we define the snippet (*i*, *j*) as a structural fragment of 9 consecutive AAs taken from the *j* − *th* template for the target starting from the AA aligned to *i*.

The snippets are then used during the reconstruction in a simulated annealing procedure [47]: at each step of the reconstruction procedure, a random snippet (*i*, *j*) is selected from the snippet library. This will replace the structure fragment [*i*, *i* + 9] in the current model, generating a new model.

The overall evaluation for the new model is then calculated through the NNPIF and processed into a single value as follows:

(5) $$P\_E_{\text{new}}=N-\left(N\times O\right)+C$$

where *O* is the output of the NNPIF (normalized from 0 to 1, where 1 is a model considered perfect and 0 a model considered completely wrong), *N* is the number of residues in the protein and *C* is a clash penalty designed to discourage the presence of couples of atoms closer than 3.73 Å.

The new model is then always accepted if the energy, *P_E_new_*, is lower than the energy for the current model, *P_E_cur_*. Otherwise, the new model is accepted with probability:

(6) $$p=e^{\left(-\Delta S/T\right)}$$

where:

(7) $$\Delta S=P\_E_{\text{cur}}-P\_E_{\text{new}}$$

and *T* is the temperature of the system, which is gradually (quadratically) lowered during the search. This allows “bad” moves to be accepted more often in the first stages of the search, so that the search will not get trapped in a local minimum early in the procedure.

The search is implemented so that an insertion will be made more frequently (80% of the times) where a coil is predicted to be.

### 2.4. Training Protocol

We propose a way of training the NNPIF in which examples are actively generated based on the search that the NNPIF directs. Although the ultimate direction of the training and composition of the training set depend on the success (and especially the lack thereof) of the NNPIF at folding protein conformations, this is essentially an instance of supervised learning, in that examples carry a label (how good, or native-like, that decoy is), which is used as a training target.

In this work, an NNPIF is initialised with random weights and used to reconstruct a number of models for a set of targets (set *S*2171). As the NNPIF aims to predict the quality for a model, a training set for NNPIF is a set of (*model*, *quality*)couples. Given that, in our experiments, we know the native structure (from the Protein Data Bank [24]) that we are trying to reconstruct, *quality* can be computed for a conformation at any stage of any reconstruction (so long as a metric for *quality* has been established), and potentially very large sets of examples can be generated. After a suitable set of examples is assembled, a first training round is performed. This same procedure can be repeated a number of times: the NNPIF trained on a set evaluates more models (by attempting a number of reconstructions), and samples from its search pathways are, in turn, added to the training set, so that a new training round can be performed.

At each round of reconstructions, the NNPIF samples a (potentially new) portion of the conformational space of *C_α_* traces. During the following training phase, the NNPIF learns more about the shape of this space. This strategy of iterative, alternate reconstruction and learning can help in obtaining an efficient sampling of such a space: a rough estimate of the energy landscape is initially obtained in the first training and is progressively enriched with details, where needed (that is, where the NNPIF leads the search), while the sampling-training cycles are carried out.

For each training, a separate test set is evaluated, composed of models coming from other trainings. The training is stopped when the error over the test set has reached a desired value.

**Figure 2:**
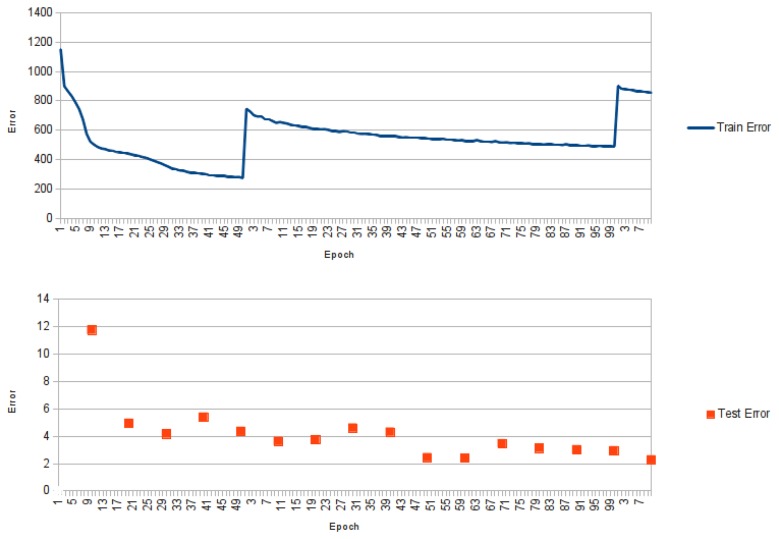
Graphic showing the training error for NNPIF over three training phases. At the end of the first training phase, new examples are generated by the reconstructor and added to the training set. This results in a spike of the error for the first epoch of the second phase. The same happens between the second and third training phase. The lower graphic shows the error for the same training over the test set, which is evaluated once every 10 epochs.

The profile of an NNPIF training is shown in Figure 2. In this case, the error for the first three cycles of training is shown against the training epochs. While the first training phase is carried out, we see a strong drop in the error. Between the first and the second training phase, new examples are added to the training set, so that when the training restarts, we have a spike in the error, partly due to the increased number of examples, partly to its increase in diversity. A similar pattern is observed between the second and third training phase and for the subsequent phases (not shown), as more and more examples are generated and added to the training set. In Figure 2, we show the error over the test set (computed every 10 training epochs). In this case, the “spiking” behaviour is much less pronounced, and an overall downwards trend is observed in the error.

Set S2171 consists in 2,171 native models (protein chains) contained in the December, 2003, version of the *pdb_select* [48], over which a 25% redundancy redundancy reduction has been performed. We used a relatively old PDB, so that we could easily gather an unrelated test set from more recent PDB versions. To keep the complexity low, only proteins of a maximum of 100 residues are chosen from the set, resulting in 382 protein chains in total.

For each target from S2171, two kinds of reconstruction are performed, backward and forward or, respectively:

- Starting from the native model;
- Starting from an unfolded, random model: each AA is randomly placed in the structure with the only constraint that the distance between two neighbours is uniformly sampled in the [3.73, 3.87]Å interval.

We use the backward reconstruction step as, otherwise, the NNPIF, especially when untrained, would be extremely unlikely to ever come close to the native structure, and this would lead to uninformative training sets containing only unfolded conformations.

Each reconstruction is run over 3,500 steps, a model perturbation (snippet insertion) at each step and evaluated by the NNPIF. For each reconstruction, a series of sample conformations from the search are taken at different intervals and added to the training set. In our tests, 50 samples are taken for each reconstruction at intervals of 10, 50 or 100 steps. If the model has not changed (all moves have been rejected) from the previous sample, the new sample is not added to the training set.

Given that the initial set is composed of 382 proteins, in a single round of reconstructions, up to 38, 200 examples are potentially added to the training set. In reality, a vast portion of these examples consists of duplicates, so just a fraction is kept (around 6, 000 in the first round of reconstructions).

With an ever-increasing training set, the training hyperparameters are kept constant through training phases, with the exception of training epochs. Training starts with an initial learning rate (fraction of the gradient of the error that is added to the free parameters) of 0.5 divided by the number of examples and kept identical over successive training phases; that is, the number of examples divider is not updated as the training set grows. The training follows a batch procedure with two examples per batch block, and as the number of examples grows between stages, so does the number of gradient steps per training epoch. The number of epochs is increased between stages: the first training phase is carried out over 50 epochs, and there is an increase of 50 epochs for each subsequent phase.

For all tests presented, the *N^F^* network has a single hidden layer with 5 units, while the *N^G^* has a single layer with 3 units. The hidden vector between *N^F^* and *N^G^* has 3 components. That is, a whole conformation is compressed into a vector of 3 real numbers. While a larger encoding may lead to a more expressive representation, a small code leads to fast evaluation times.

### 2.5. Clustering and Model Selection

For the clustering phase, we use an implementation of the DBSCANalgorithm [49]. This density-based clustering algorithm has some characteristics that make it fit for our purpose. It does not need any knowledge about the number of clusters in the data, and it contains an implicit notion of noise. The clustering process is based on the concept of density reachability, and it is guided by two parameters: the first, generally referred to as *ε*, is the minimum similarity that two points need to have to be associated with the same cluster; the second is the minimum number of points required for a cluster being formed. Given the matrix, M, containing the GDT-scores between each pair of models, *M*(*i*, *j*) = *GDT*(*model_i_*, *model_j_*), different configurations of clusters can be obtained by varying the two parameters. We selected five models by adjusting the parameters in order to obtain a configuration with five or more clusters, with the largest cluster containing at least 20 models. For each of the five largest clusters, a centroid is selected by picking the model with the highest sum of GDT-scores against the other models that belong to the same cluster. The centroids represent the selected models for the target protein.

## 3. Results and Discussion

We have performed two tests in order to measure the accuracy of the NNPIF-based predictor, using two different test sets containing a number of target proteins (targets).

The first test focuses on measuring the accuracy of the NNPIF in predicting the quality of the models while they are being reconstructed. In order to isolate the performance of the NN from the other variables in the system, 1D feature predictions in the first place, we use exact 1D features for each of the target proteins (secondary structure, solvent accessibility, structural motifs, *etc*.) when building the snippet library. This way, we evaluate the NNPIF's performances independently of 1D feature prediction accuracy.

The set for the first test is composed of 21 protein chains of less than 100 residues in length (S20). This is obtained by running a 30% redundancy reduction on the September, 2007, version of the PDB against the December, 2003, version of the PDB [24]. The 21 protein chains are randomly selected from the resulting set. It is worth noting that the snippet libraries in this case may contain, among others, snippets from the native structure for the targets, as the PDB used to extract them is the same from which the targets have been picked. However, this does not impede an objective evaluation of the performance of the NNPIF, as the snippets are randomly selected during the model reconstruction procedure, the NNPIF has been trained on an older PDB from 2003, and especially, the reconstruction does not contain any constraint from templates other than, possibly, the very short range ones included in the snippets.

The second test focuses on gauging the competitiveness of our system when compared to other *ab initio* predictors that have taken part in the last three editions of CASP [50]. In this case, we have put ourselves in a CASP-like scenario, where we use just the primary structure for the targets, predict the 1D features to build the snippet database before the reconstruction of the models is carried out and use a PDB version pre-dating the test proteins.

Only three mono-domain *ab initio* (or *Free Modeling*) targets with less than 100 residues in length have been released in the last three CASP competitions (CASP8, CASP9, CASP10). It would be unfair to perform tests on Free Modellingdomains that part of multi-domain proteins, as those are not necessarily modelled separately by predictors at CASP. Likewise, a comparison would not be fair if we tried to predict targets from the *Homology Modeling*(or *Template-Based Modeling*) category or targets from older CASP editions, as our system is purely *ab initio*. We only test on short targets, as no decoys longer than 100 residues were used for training.

When a target is evaluated, an initial, unfolded model is built by placing the first atom of the *C_α_* trace at coordinates [0, 0, 0]. Random coordinates are then generated for the subsequent atoms, with the only constraint that the distance between this atom and its predecessor is uniformly sampled in the [3.73, 3.87]Å interval. For each target protein in the set, 1,000 models are reconstructed with 100,000 snippet insertion attempts per reconstruction. Each of the reconstructions start with a different seed for the random number generator, that is, it starts from a different conformation and follows a different snippet substitution procedure. The models are then clustered based on their structure. We select five models per target protein, as we would do in a CASP competition; so, the cluster centroids for the top five clusters are selected as candidate structures.

**Table 1:** Results of the test with the first dataset to gauge the accuracy of the NNPIF. For each target, we show its PDB (Protein Data Bank) ID and length. We show Global Distance Test (GDT) scores for the best model, the average GDT over all of the models, the GDT for the best of the five final models selected via clustering and the RMSD for the best model and the average of the mutual GDT scores between the 1,000 reconstructed models. Avg., average; Clust., clustering.

**Id.**	**Length**		**GDT Max**	**GDT Avg.**	**GDT Clust.**	**RMSDbest**	**m**
2E5TA	46	0.4457	0.3651	0.4022	17.10	0.4942
2EKFA	61	0.3975	0.3073	0.3975	10.17	0.3782
2K0NA	85	0.3824	0.2709	0.3	6.83	0.2891
2PQRC	40	0.5125	0.3792	0.4125	4.91	0.4701
2QFAB	62	0.5202	0.3853	0.4556	5.58	0.4269
2QKHA	94	0.2926	0.2084	0.2394	9.92	0.2458
2QZGA	88	0.3239	0.228	0.2528	14.83	0.2742
2VC8A	72	0.3854	0.2689	0.3021	7.13	0.2801
2YVRA	45	0.4722	0.3613	0.4	5.40	0.4267
3BRTB	61	0.4016	0.3071	0.3607	18.87	0.3921
1BDSA	45	0.5233	0.3776	0.4709	4.01	0.4031
1BH8A	47	0.5944	0.433	0.5111	4.61	0.4338
2EQFA	48	0.4674	0.3322	0.3804	5.22	0.3815
2JEEA	80	0.4423	0.3116	0.3686	6.64	0.368
2JP7A	59	0.5088	0.3369	0.4211	5.36	0.3688
2P9XA	100	0.3316	0.2186	0.2423	10.04	0.2353
2R9IA	73	0.5486	0.4071	0.4514	10.98	0.4336
2V6YA	77	0.4433	0.3009	0.3367	7.31	0.3132
2YQPA	62	0.4583	0.3311	0.3958	10.42	0.3505
2YRMA	45	0.4593	0.3399	0.4128	8.94	0.3618
3CJHA	63	0.5962	0.3908	0.4279	4.53	0.4022

We show the results for the first test in Table 1 and the results for the second test in Table 2. For each target, we show its PDB ID and length. The scores are GDT scores and RMSDcalculated with the TMscoreprogram [51]. We show the score for the best model (“GDT Max”), the average GDT over all of the models (“GDT Avg.”), the GDT score for the best of the five final models selected via clustering (“GDT Clust.”) and the RMSD between the best model and the native structure.

**Table 2:** Results of the test with the second dataset to gauge the accuracy of the ab initio predictor. For each target, we show its PDB ID and length. We show GDT scores for the best model, the average GDT over all of the models, the GDT for the best of the five final models selected via clustering, the RMSD for the best model and how the best between the selected models would have ranked at CASP in the “server” category.

**Id.**	**Length**	**GDT Max**	**GDT Avg.**	**GDT Clust.**	**RMSD best**	**CASPRank**
T0531-D1	67	0.3966	0.2763	0.3966	6.35	6 over 310
T0624-D1	69	0.3659	0.2656	0.3406	7.89	91 over 320
T0476-D1	88	0.2874	0.1965	0.227	15.16	189 over 286

In the last column of Table 2 (“CASP Rank”), we show how the top model between the five models selected via clustering would have ranked against all of the models submitted at CASP (CASP8 for T0476-D1 [52], CASP9 for T0531-D1 [53] and T0624-D1 [54]) in terms of the GDT score. As this is not a human predictor, we exclude from the ranking models from competitors of the “human” category, so only the models submitted from automated servers are considered.

It is worth noting that all of the GDT scores for our models have been calculated by superimposing just the predicted *C_α_* trace to the native structure, rather than the full atom model. This gives us a good approximation of the quality of the reconstructions, but the score could change marginally if side-chains were added. Only full atom models are submitted to the CASP competition, so the “virtual ranking” given in Table 2 is not entirely accurate, but still indicative.

In 15 cases out of 21 proteins in the first test, the GDT score of the best model is above 0.4 (in 7 above 0.5). The clustering algorithm always allows one to pick at least a model whose GDT against the native is above the average of the reconstructed models. In two cases, it was possible to select the best from the reconstructed models.

**Figure 3:**
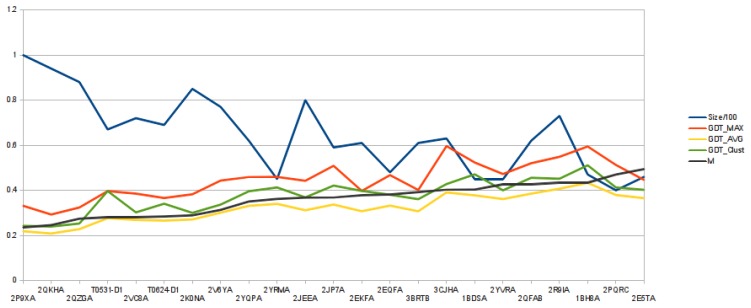
Graphic showing the relation between the average over the mutual GDT matrix (*m*) and the quality of the reconstructed models.

One-thousand models (*C_α_* traces) are reconstructed for each target, and those are then clustered based on their similarity. Consequently, any model from a cluster is structurally more similar to any other model from the same cluster than any other model from any of the other clusters, as clusters are obtained by superimposing any two of the 1,000 models and calculating their mutual GDT score in the similarity matrix, *M*. From Figure 3, we can observe that the average quality of the reconstructed models follows the same trend as number *m*, defined as:

(8) $$m=\frac{1}{N^{2}}\sum_{i=1}^{N}\sum_{j=1}^{N}M(i,j)$$

In the Figure, for each target, its length (divided by 100), GDT Max, GDT Avg., GDT Clust. and *m* are plotted.

This suggests that when the NNPIF is more effective in distinguishing a good model from a bad one (higher scoring models are reconstructed), the resulting reconstructed models will be, on average, more similar to each other. The NNPIF implements a somewhat arbitrary model of a protein's energy landscape, as it is trained using a purely geometrical target. Reconstructing a protein entails looking for the minimum of this energy landscape. In an ideal case, the energy landscape would resemble a funnel with a global, deep minimum that is the native conformation [55]. For the NNPIF, the energy landscape model is not perfect and is not a deep funnel with a global minimum, but a noisy surface with many local minima and a global minimum that is both hard to reach and not coinciding with the native state. The graph in Figure 3 suggests that the more the NNPIF's landscape is funnel-like, the closer its minimum is to the one in the true energy landscape. When we obtain models that are close to the native one, those are also more similar to each other. Where the energy landscape is more flat and noisy, the models diverge structurally both from the native and between each other. This means that an estimate of the quality of the models can be obtained by looking at how structurally close they are.

While the GDT score alone is not enough to determine the quality of a model, it is possible to evaluate the results by a visual comparison between the native structure and the best among the five models selected via clustering.

**Figure 4:**
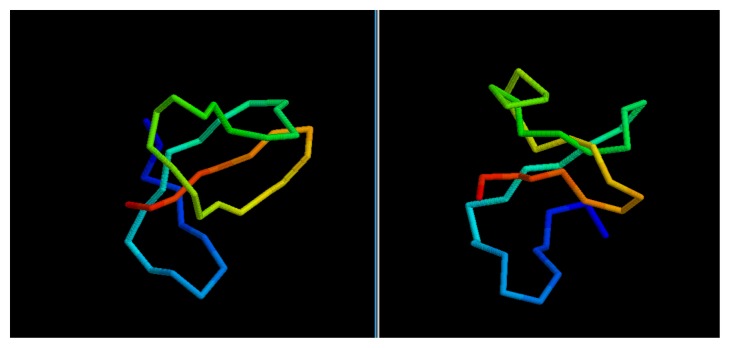
Comparison between native target 1BDSA and the best of the selected reconstructed models.

**Figure 5:**
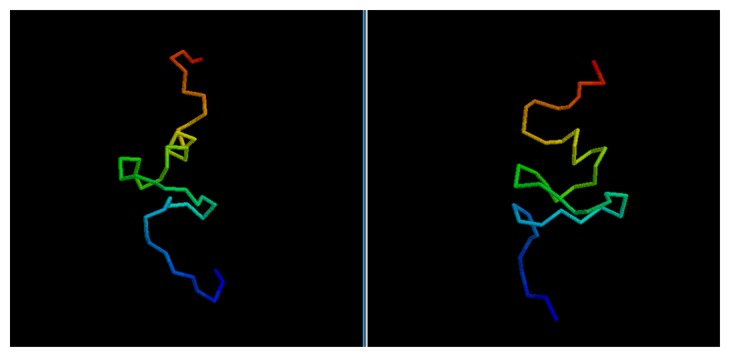
Comparison between native target 2EQFA and the best of the selected reconstructed models.

**Figure 6:**
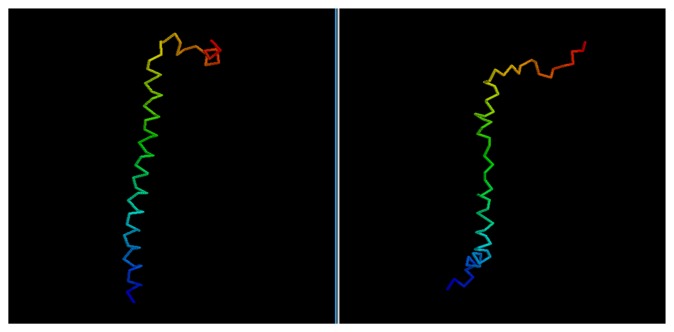
Comparison between native target 2QFAB and the best of the selected reconstructed models.

**Figure 7:**
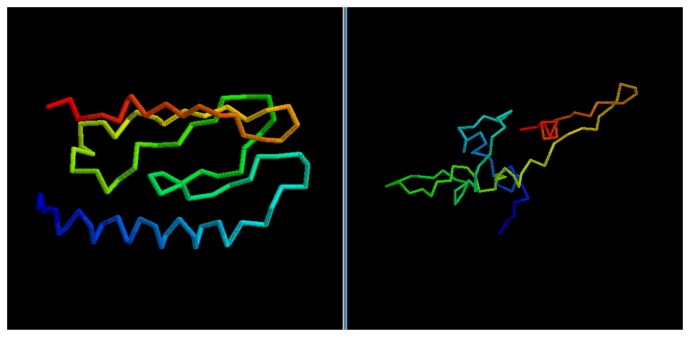
Comparison between native target 2QKHA and the best of the selected reconstructed models.

**Figure 8:**
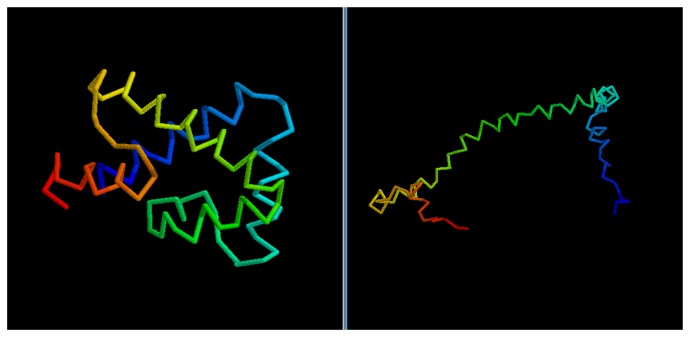
Comparison between native target 2P9XA and the best of the selected reconstructed models.

In most cases, we can see how the topology of the model is close to the native one: this is the case for targets 1BDSA (Figure 4), 1BH8A, 2EKFA, 2EQFA (Figure 5), 2JP7A, 2PQRC, 2QFAB (Figure 6), 2YQPA, 2YVRA and 3CJHA.The best of the selected models for these proteins have a GDT Score ranging from 0.38 to 0.51. In other cases, the topology is locally correct, but the long-distance interactions are not predicted correctly. One example of this is the model of target 2QKHA (Figure 7; GDT score: 0.23), where the *β* — *sheet* has been only partially packed, resulting in the reconstruction of three separated couples of strands, rather than a single, six-stranded sheet. Only for three targets, the NNPIF was completely unable to approximate the correct folding, and that always resulted in a roughly unfolded model; this is the case for targets 2QZGA, 2P9XA, 2JEEA. Looking at the best selected model for target 2P9XA (Figure 8; GDT score: 0.24), it is possible to see that most of the central part of the structure has been predicted as a long, stiff helix. The main problem in this case is the presence of the wrong kind of snippets in a given position of the snippet library. If a coil connecting two helices is predicted (by the secondary structure predictor) to be a helix instead, the snippets selected for that position will generally be helices, as well. Interestingly, and not entirely surprisingly, all of the completely misfolded proteins are among the longest ones (88, 100 and 80 AAs, respectively). Although longer proteins are intrinsically harder to fold, it is also possible that the NNPIF has been trained with too few examples of long protein chains.

It is also evident from visual inspection that the NNPIF only returns “sketches” of proteins rather than physically valid models. This is not surprising, as it implements a potential that is not modelled on physics, and especially, it only reconstructs *C_α_* traces. These models may benefit from a physics-based refinement, which may not be the case for models obtained from a more refined initial protocol that already incorporates physical information and a richer representation of a protein conformation.

**Figure 9:**
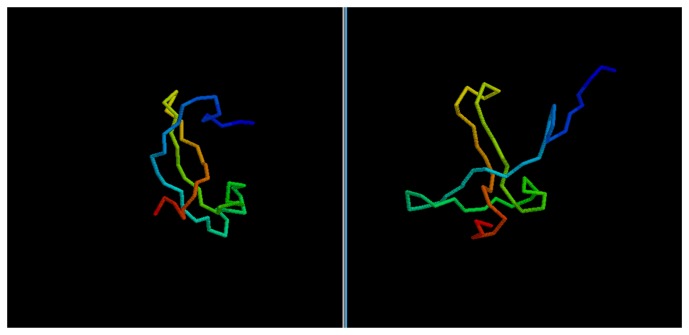
Comparison between native target T0531-D1 and the best of the selected reconstructed models.

We obtain similar results if we consider the CASP targets (Table 2). The predictor fails in properly folding the longest target, T0476-D1. This would have resulted in a low ranking in the CASP8 competition among the servers (though T0476-D1 was not a pure *Free Modelingtarget*; hence, templates could be found for it and the prediction likely improved with them). On the other hand, for shorter proteins, the quality of the selected models is significantly higher and, so, the virtual CASP ranking: target T0624-D1 would have ranked in the top third of the CASP server ranking, while target T0531-D1 (Figure 9) peaks as the overall sixth best model in the server category.

An NNPIF-based predictor, complemented by a refinement stage, may be a good *ab initio* challenger in the next CASP experiment, especially if it is retrained with a larger dataset containing longer protein chains. The small number of longer proteins in the training set causes a bias towards the prediction of short-distance interactions, which may be obviated by modifying the training set composition.

## 4. Conclusions

In this article, we have introduced an *ab initio* reconstructor of protein structures, guided by a knowledge-based evaluation function implemented as a class of neural networks that we have designed: NNPIF. The evaluation function is trained on sets of decoys, which are iteratively generated by sampling reconstruction pathways induced by the function itself. This allows us to selectively sample the conformational space where the evaluation function is likely to explore it. The training-reconstruction protocol iterates until we observe a plateau in performances. While the NNPIF is a simple algorithm that evaluates a very basic representation of a protein (its *C_α_* trace), it is capable of accommodating extensive contextual information in its inputs, as several neighbouring amino acids (both along the sequence and in the Euclidean space) can be considered when evaluating the interaction between two atoms. Although *ab initio* results are generally hard to assess, and the tests we present here are somewhat preliminary, we observe promising performances on a set of short proteins, with a majority of them predicted at better than random levels (15 out of 21 test cases with GDT scores above 0.4) and one of three CASP postdictions being state-of-the-art. We also observe that, when the evaluation landscape induced by the NNPIF is funnel-like, the endpoints of the reconstructions tend to be closer to the native structure. Although we have no evidence that suggests that, in these cases, the evaluation landscape is closer to the true energy landscape (other than its minimum), this can be used to guide model selection. In this article, we have presented a clustering procedure that allows us to select better-than-average models in all cases and top models in some cases.

It is also possible to design a protocol based on the reinforcement learning paradigm [56] in which samples become simple stages on the path towards a final folded conformation, and the quality of this conformation only is used to evaluate the whole path, similarly to [27]. This paradigm might be advantageous in a number of ways: for instance the choice of a metric for native-likeness would become less critical, as only the end result of a search is explicitly evaluated, rather than all its stages; probably more importantly, a locally non-native-like conformation might be still scored positively if paths leading through it tend to yield good solutions. In essence, this paradigm would automatically induce a folding landscape, while by scoring each decoy separately, we are effectively choosing (possibly arbitrarily) the target folding landscape: a conformation that is geometrically different from the native is “bad”, even if it may be a useful stage in the folding process. In other words, a reinforcement learning protocol might shed some light on the folding process, by ranking folding pathways rather than individual decoys. However, its computational cost would have likely been much larger, and we are considering this as a future direction to explore, alongside the use of more extensive training sets, including larger proteins, as the tests presented in this work are limited to proteins containing at most 100 residues.

Another direction we are considering is the exploration of simple refinement protocols for the endpoints of the reconstructions. The NNPIF reconstruction protocol produces guesses that are noticeably non-physical. Naively adding all the backbone atoms and side chains leads to conformations largely devoid of hydrogen bonds and containing steric clashes and a refinement procedure that, minimally, drives the formation of the former, and the elimination of the latter may lead to improved models.
